# Class of hemorrhagic shock is associated with progressive diastolic coronary flow reversal and diminished left ventricular function

**DOI:** 10.3389/fphys.2022.1033784

**Published:** 2022-12-14

**Authors:** Noha N. Elansary, David P. Stonko, Rebecca N. Treffalls, Hossam Abdou, Marta J. Madurska, Jonathan J. Morrison

**Affiliations:** ^1^ R. Adams Cowley Shock Trauma Center, University of Maryland Medical System, Baltimore, MD, United States; ^2^ Department of Surgery, University of Maryland Medical System, Baltimore, MD, United States; ^3^ Division of Vascular and Endovascular Surgery, Department of Surgery, Mayo Clinic, MN, United States

**Keywords:** hemorrhagic shock, pressure-volume (P-V) loop, coronary artery flow, left ventricular function (LV function), exsanguination cardiac arrest

## Abstract

**Introduction:** The relationship between coronary artery flow and left ventricular (LV) function during hemorrhagic shock remains unknown. The aim of this study was to quantify coronary artery flow directionality alongside left ventricular function through the four classes of hemorrhage shock.

**Methods:** Following baseline data collection, swine were exsanguinated into cardiac arrest *via* the femoral artery using a logarithmic bleed, taking each animal through the four classes of hemorrhagic shock based on percent bleed (class I: 15%; class II: 15%–30%; class III: 30%–40%; class IV: >40%). Telemetry data, left ventricular pressure-volume loops, and left anterior descending artery flow tracings over numerous cardiac cycles were collected and analyzed for each animal throughout.

**Results:** Five male swine (mean 72 ± 12 kg) were successfully exsanguinated into cardiac arrest. Mean left ventricular end-diastolic volume, end-diastolic pressure, and stroke work decreased as the hemorrhagic shock class progressed (*p* < 0.001). The proportion of diastole spent with retrograde coronary flow was also associated with class of hemorrhagic shock (mean 5.6% of diastole in baseline, to 63.9% of diastole in class IV; *p* < 0.0001), worsening at each class from baseline through class IV. Preload recruitable stroke work (PRSW) decreased significantly in classes II through IV (*p* < 0.001). Systemic Vascular Resistance (SVR) is associated with class of hemorrhage shock (*p* < 0.001).

**Conclusion:** With progressive classes of hemorrhagic shock left ventricular function progressively decreased, and the coronary arteries spent a greater proportion of diastole in retrograde flow, with progressively more negative total coronary flow. Preload recruitable stroke work, a load-independent measure of inotropy, also worsened in severe hemorrhagic shock, indicating the mechanism extends beyond the drop in preload and afterload alone.

## Introduction

Exsanguination cardiac arrest caused by hemorrhagic shock is one of the leading causes of death following a traumatic injury (Kauvar and Wade, 2005; Morrison and Rasmussen, 2012; Kisat et al., 2013; Morrison, 2017; Patel et al., 2022a). Many interventions aim to combat hemorrhagic shock; however, the prognosis is still poor, reaching 20%–30% for non-compressible torso hemorrhage.3 While hemorrhagic shock and the body’s compensatory mechanisms have been extensively studied, changes in coronary flow and the relationship to left ventricular function at each class of hemorrhagic shock remain unquantified. This is partially due to the difficulty of reliably recording coronary flow in experimental settings and because it is impractical to obtain clinically (Ootaki et al., 2008; Sakata et al., 2021; Stonko et al., 2022a).

The myocardium has a relatively high oxygen consumption compared to other organs, with a 70%–80% extraction percentage compared to skeletal muscles, which have a 30%–40% oxygen extraction. (Braunwald, 1971; Kemppainen et al., 2002) The increased oxygen extraction makes the heart especially dependent on blood delivery and, in turn, makes the coronary vascular tone important to myocardial perfusion (Merkus et al., 2002; Goodwill et al., 2017; Heusch, 2022). Coronary flow is regulated through multiple mechanisms such as coronary perfusion pressure (CPP), extravascular compressive forces, myogenic, and neural and hormonal influences.

Coronary arterial flow is phasic, with the compressive forces during systole counteracting forward flow in the coronary artery and may result in periods of retrograde coronary flow. (Ferro et al., 1995; Ramanathan and Skinner, 2005) Consequently, the myocardium is mainly perfused during diastole, which is when the left ventricle receives 80% of its blood volume. Without the compressive forces, the heart would be perfused during all classes of the cardiac cycle. There is, furthermore, a complex relationship between CPP and coronary autoregulation, which allows the coronary arteries to match myocardial demand over a range of approximately 60–180 mmHg in humans (Feigl, 1989). Here, vasoconstriction will improve coronary flow when CPP is low, but this reverses when CPP is elevated (Duncker and Bache, 2008).

Coupled LV pressure-volume (PV) loop analysis and direct coronary artery flow measurements during hemorrhage would provide a mechanism for better understanding the effect of hemorrhage on cardiovascular physiology. Stonko et al. (2022a) previously showed that, compared to baseline, hemorrhagic shock was associated with a reduction in end-systolic pressure (ESP) and volume (ESV), as well as stroke work (SW), elastance (Ea), and contractility (Ees). This study also showed that shock was associated with increased time of coronary flow reversal–that during severe hemorrhagic shock, the coronary arteries spent up to 30% of the cardiac cycle in retrograde flow. Stonko et al., 2022a These coronary flow dynamics were thought-provoking as they may be a potential therapeutic target. However, a further assessment of this was limited because the study was not constructed to facilitate a detailed understanding of how left ventricular function and coronary flow change as a patient evolves from baseline through increasingly severe classes of hemorrhagic shock, and it did not examine diastole in particular, which is the critical period in which the LV receives flow. Therefore, this study aimed to characterize the changes in cardiovascular physiology (specifically, LV function and coronary artery flow direction during diastole) through the four classes of hemorrhagic shock using a swine model of exsanguination cardiac arrest.

## Materials and methods

### Study overview

The study was conducted at the University of Maryland School of Medicine, accredited by the American Association for Laboratory Animal Science. The Institutional Animal Care and Use Committee (IACUC) approved the protocol. The study utilized five adult male Yorkshire swine (*Sus scrofa*), prospectively enrolled for specifically this study, weighing between 70 and 80 kg. We included all five of the five animals that underwent successful exsanguination to cardiac arrest and experienced all four classes of hemorrhagic shock and did not receive vasoactives for which coronary flow data was available. Species selection and sample size calculation were computed based on a literature review with a qualified IACUC librarian with preliminary data from prior work, identifying coronary flow reversal during hemorrhagic shock. (Stonko et al., 2022a; Charan and Kantharia, 2013) For acclimatization, animals were housed singly in cages within larger communal pens to allow the animals to interact, in the animal facility under the care of licensed veterinary staff, with free access to food and water for a minimum of 72 h before experiments. Animals were fasted for 12 h before experiments in preparation for procedures. This indoor facility had 12-h on/off light cycles, and experiments started uniformly at approximately 7:00 a.m. and lasted for approximately 5 h.

The study consisted of two overall phases: animal instrumentation and a controlled exsanguination phase. At the end of the protocol, all animals were euthanized.

## Animal protocol

### Animal instrumentation and monitoring

Animals were sedated with an injection of Telazol (4.5 mg/kg) and Xylazine (2 mg/kg) intramuscularly. To induce general anesthesia, isoflurane was administered *via* facemask, followed by orotracheal intubation. A warming blanket was used to prevent hypothermia during the procedure. Animals were mechanically ventilated with 2% isoflurane, targeting a 40% fraction of inspired oxygen, tidal volumes of 7–10 ml/kg, and with 10–15 breaths per min and adjusted as appropriate based on arterial blood gases (ABGs) every 15 min. A warming blanket set at 39°C was used to prevent hypothermia.

Ultrasound (Lumify, Phillips, Eindhoven, Netherlands) guided Seldinger technique was used to obtain percutaneous vascular access. Under fluoroscopic guidance, a cardiac PV loop catheter (Transonic Corporation, Ithaca, NY) was introduced into the left ventricle, and three solid-state pressure catheters (Transonic Corporation, Ithaca, NY) were positioned in the right ventricle, right atrium, and aortic arch *via* the neck vessels. We have previously described technical and surgical details for using PV loop catheters in swine (Edwards et al., 2021a; Stonko et al., 2021; Stonko et al., 2022b; Madurska et al., 2022). Vascular access to the right femoral artery was obtained to permit controlled exsanguination.

A mini-laparotomy and urinary cystotomy were performed for urinary drainage, along with a splenectomy to prevent autotransfusion. An anterolateral left thoracotomy with left coronary dissection was performed to place a 3 mm perivascular flow probe (Transonic Corporation, Ithaca, NY) on the left anterior descending (LAD) coronary artery.

**FIGURE 1:**
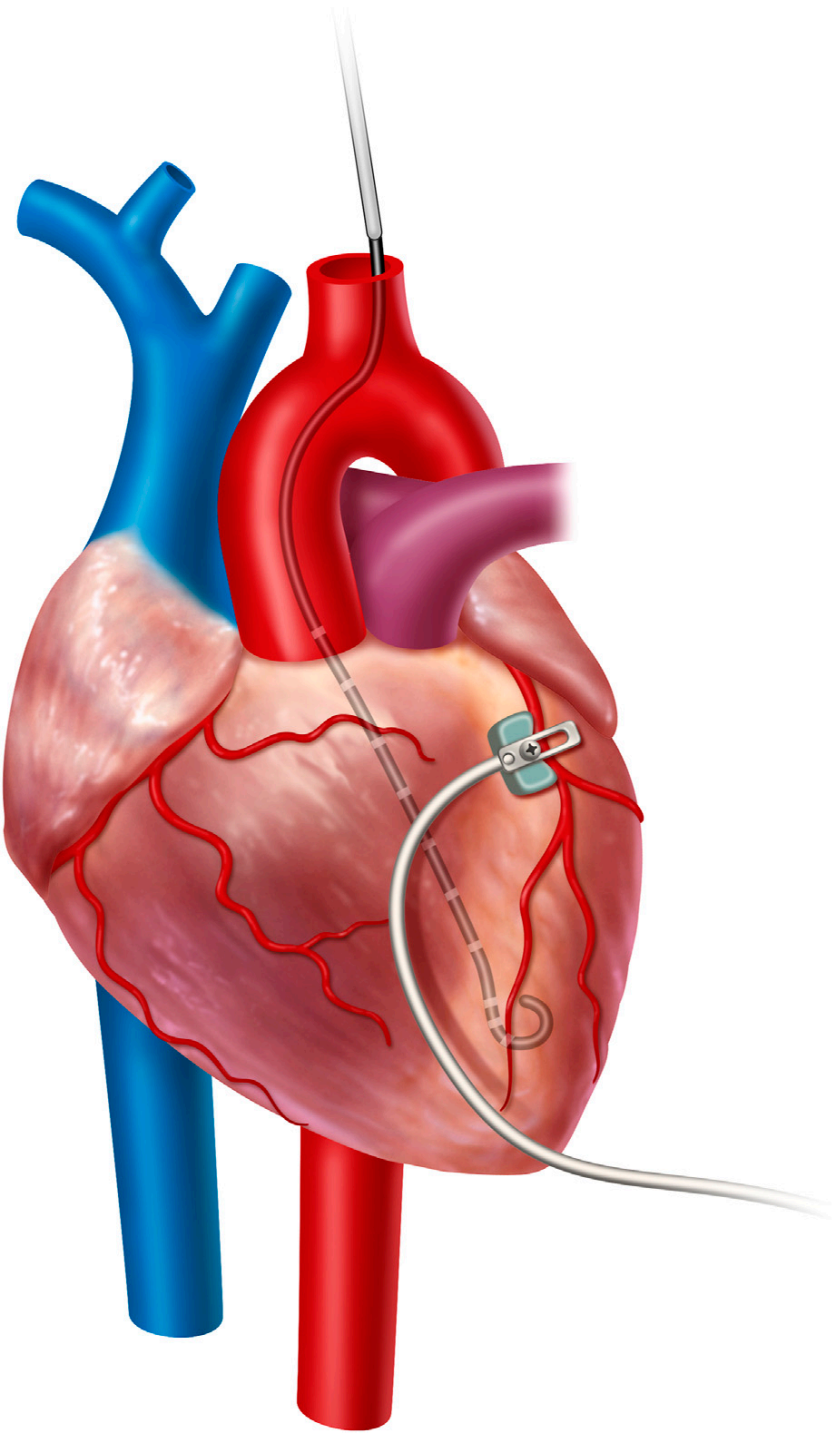
Cartoon representation of the central instrumentation, with a PV loop in the left ventricle and the dissected left coronary artery with LAD coronary flow probe placement. Not shown is a peripherally inserted, solid-state pressure catheter for central venous pressure monitoring and a peripherally inserted solid-state pressure catheter for arterial blood pressure monitoring.

The locations of the pressure and PV loop catheters were confirmed with C-arm fluoroscopy (OEC 9800, General Electric, Boston, United States) and direct palpation. The animals were monitored using electrocardiography (ECG), temperature probes, pulse oximetry, and pressure tracings from the pressure catheters. Figure 1 shows a cartoon representation of the central instrumentation, with a PV loop in the left ventricle and the dissected left coronary artery with LAD coronary flow probe placement. Not shown is a peripherally inserted, solid-state pressure catheter for central venous pressure monitoring. A peripherally inserted solid-state pressure catheter is also in the aorta to measure arterial blood pressure.

### Exsanguination

After a baseline period of 15 min was observed to allow physiologic equilibration and baseline data capture, a baseline ABG was drawn. Animals were administered 30,000 units of heparin intravenously, followed by controlled, logarithmic exsanguination, as previously described (Madurska et al., 2021), until asystole. Asystole was determined by electrical activity on the ECG. Blood volume was determined by an assumption of 66 ml/kg for adult swine. (Edwards et al., 2021a; Swindle and Smith, 2016; Bush et al., 1955; Abdou et al., 1097) The target percent blood loss was 50% at 45 min and was achieved using a pre-programmable peristaltic pump through the femoral access sheath to achieve exsanguination cardiac arrest. The blood was weighed using a tared scale to confirm the volume of hemorrhage from the calculated flow from the pump. If there were any discordance or uncertainty, the volume of blood would be confirmed with a graduated cylinder. Using this tightly controlled rate of bleeding, class of hemorrhagic shock (class I: 15%; class II: 15%–30%; class III: 30%–40%; class IV: >40%) may be correlated based on the precise blood loss with the animal’s physiologic state with the PV loop analysis and the coronary flow monitor.

## Data and statistical analysis

### Data capture and analysis

Animal weight and exsanguinated blood volumes were recorded. Physiological data were continuously collected using an integrated life science data acquisition system (PowerLab and LabChart, ADInstruments, Sydney, Australia). All data were exported to Microsoft Excel (Redmond, WA) for analysis, PV loop data was exported to MATLAB for analysis (Stonko et al., 2022c; Stonko et al., 2022d) (Mathworks, Nantick, MA, United States), and coronary flow data was exported to and analyzed in Python (Van Rossum and Drake, 1995). Physiologic data were captured and analyzed based on the four classes of shock as described above. Here (Leach and Treacher, 2002), seconds of representative coronary waveforms, central pressures and ECG tracing are captured according to percent blood loss when the animal entered hemorrhagic shock and these same segments are analyzed, with the first 0.5 s being plotted graphically. These data were visualized with GraphPad Prism (San Diego, California). Each animal’s data were averaged and graphed as a single data point for the hemodynamic parameters.

### Myocardial oxygen consumption

Hemoglobin was sampled at baseline and end of the study for each animal. A non-linear regression analysis was performed to interpolate hemoglobin values for each animal for each class of hemorrhagic shock. Myocardial oxygen consumption (DO2) was then calculated by the following formula, DO2 = CF × [1.39 × (Hb) × SaO2 + (0.003 × PaO2)] where [Hb] was derived by this interpolation.30 Data were compared from baseline to each class of hemorrhagic shock using a 2-way ANOVA with multiple comparisons accounted for with Tukey’s method.

### PV loop and coronary flow analysis

LabChart analyzes each cardiac cycle and outputs hemodynamic parameters. The parameters include stroke work (SW), heart rate, and measures of preload, afterload, and contractility over time. In a previous study, we described the methodology for analyzing PV loop data over time to output a single PV loop representing LV function during that period and published Matlab code to execute this procedure (Stonko et al., 2022c; Stonko et al., 2022d). This facilitates the computation of the end-systolic pressure-volume relationship (ESPVR) and end-diastolic pressure-volume relationship (EDPVR). From the averaged PV loop, other average hemodynamic measures can be determined, including cardiac output, end-systolic pressure (ESP), end-diastolic pressure (EDP), end-systolic volume (ESV), end-diastolic volume (EDV), as well as ejection fraction (EF), cardiac output (CO), stroke work (SW) and volume (SV) and arterial elastance (Ea).

Coronary flow was also exported from LabChart into Python. The coronary flow and PV loop parameters were compared from baseline to each class of hemorrhagic shock using 2-way ANOVA with multiple comparisons accounted for with Tukey’s method. Coronary vascular resistance (CVR) was also calculated using the following formula: coronary perfusion pressure (CPP)—right atrial pressure (RAP)/coronary flow. CVR was compared from baseline to each class of hemorrhagic shock using 2-way ANOVA with multiple comparisons accounted for with Tukey’s method. Preload recruitable stroke work (PRSW) was used to measure left ventricular function; a simple linear regression was performed by graphing SW versus EDV for each class of hemorrhagic shock. A Goodness of Fit test was conducted on each line. Slopes and intercepts were compared for each category with 2-ANOVAs, and multiple comparisons were accounted for with Tukey’s method. Both PV loop parameters and coronary flow metrics were calculated and plotted as means with standard deviations.

## Results

**FIGURE 2:**
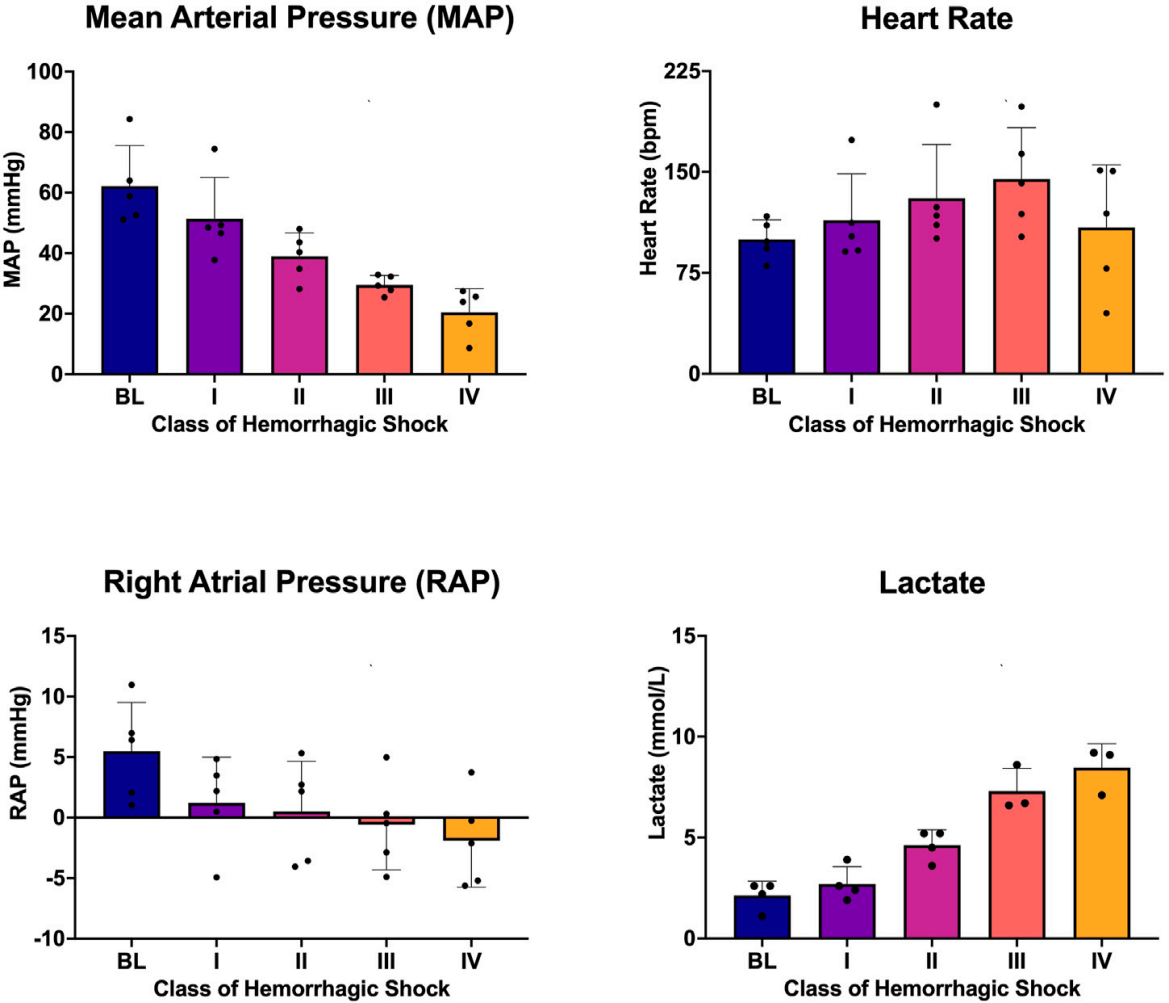
Mean arterial pressure (MAP), heart rate (HR), right atrial pressure (RAP), and whole blood lactic acid at each class of hemorrhagic shock (I-IV) induced by this logarithmic hemorrhage.

**FIGURE 3:**
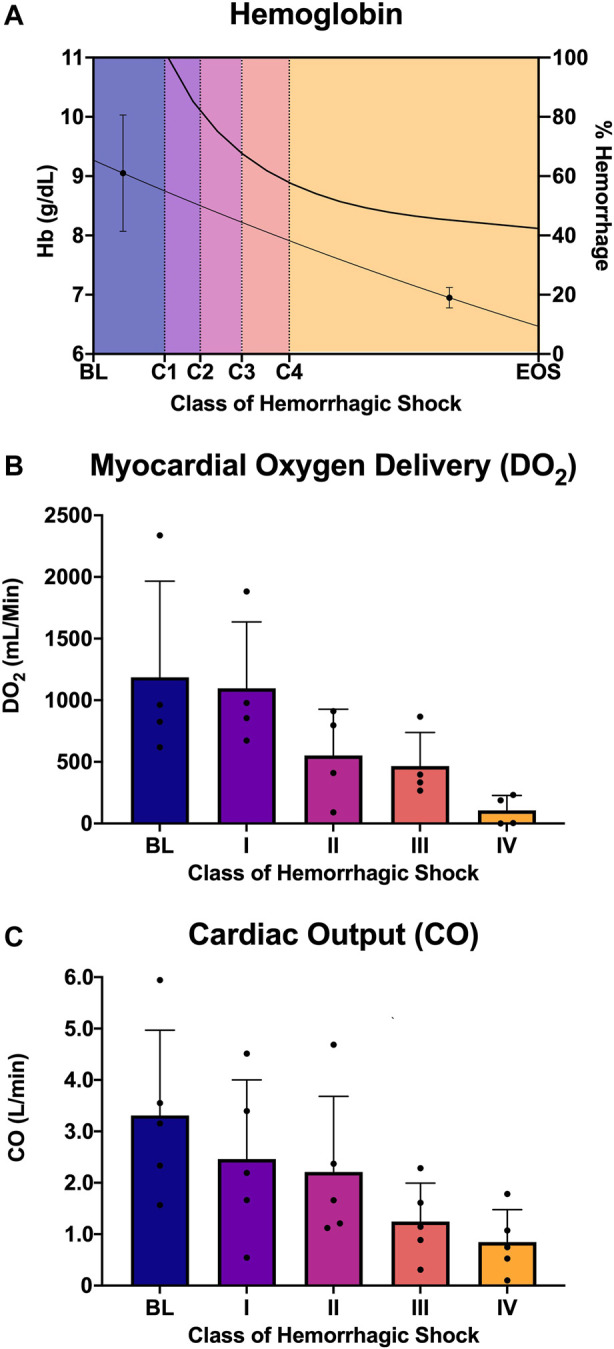
Analysis of oxygen delivery to the heart: **(A)** Hemoglobin and estimated percent of blood hemorrhaged from the logarithmic bleed at each study period, **(B)** myocardial oxygen delivery (DO2) from baseline and each class of shock, and **(C)** cardiac output from baseline and each class of shock.

Five animals with a mean weight ± standard deviation of 72 ± 12 kg were enrolled in the study. Hemorrhagic shock was induced, and animals were hemorrhaged into exsanguination cardiac arrest. All animals survived to class IV of hemorrhagic shock. Mean arterial pressure (MAP), heart rate, right atrial pressure (RAP), and whole blood lactic acid were sampled during the progression from baseline through class IV hemorrhagic shock, and all were associated with the class of hemorrhagic shock (*p* < 0.0001; Figure 2). Hemoglobin and cardiac output (CO) were also sampled and were associated with the class of hemorrhagic shock (*p* < 0.0001, Figures 3A, C). Myocardial oxygen delivery (DO2) was calculated and also found to decrease with the class of hemorrhagic shock, and it differs significantly between baseline and class III and IV (*p* < 0.0001; Figure 3B). Before asystole, no cardiac arrhythmias other than sinus tachycardia were noted during the experiment or on *post hoc* assessment of telemetry data. The mean time to asystole was 53 ± 15 min, and an average of 52 ± 11% of the total blood volume was lost by the end of the experiment.

### Left ventricular functional changes during hemorrhagic shock

**FIGURE 4:**
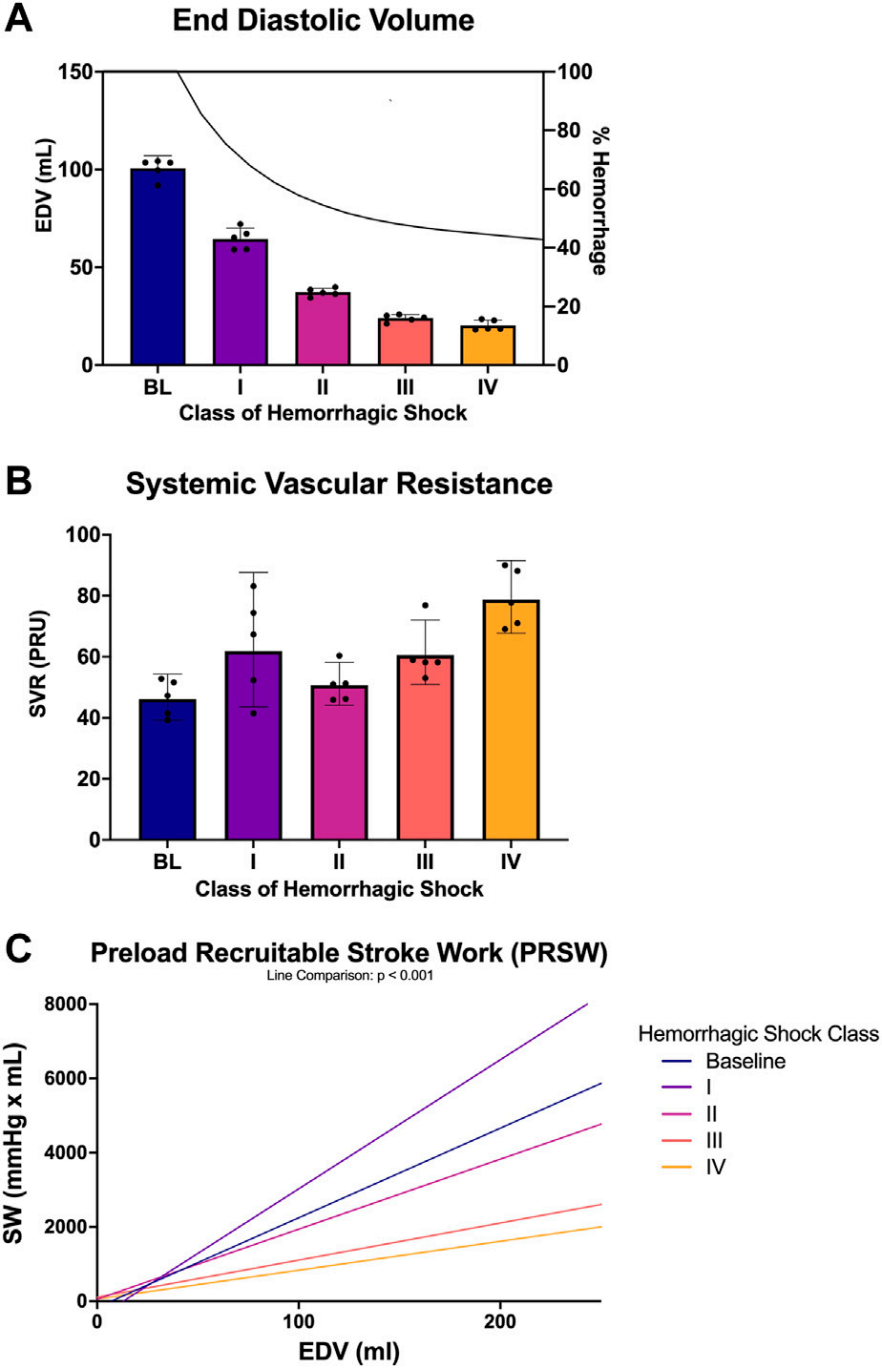
PV loop analysis across baseline and each class of hemorrhagic shock **(A)** end-diastolic volume (EDV) and estimated percent blood hemorrhaged from the logarithmic bleed at each study period, **(B)** systemic vascular resistance (SVR) from baseline and each class of shock, and **(C)** preload recruitable stroke work (PRSW) from baseline and each class of hemorrhagic shock.

PV loops over numerous cardiac cycles were collected for each animal from baseline through each class of hemorrhagic shock and examined. Figure 4A shows representative PV loops for one animal from each class of the study. Here, the PV loops migrated down and to the left within the PV plane, but qualitatively, within the bounds of the end-systolic and end-diastolic pressure-volume relationship (ESPVR and ESDVR). ESP and ESV decreased, as did EDP, EDV, and stroke volume. Stroke work (the area within the PV loop) also decreased with each class of hemorrhagic shock. The other four animals’ PV loop progression through the classes of hemorrhagic shock are presented as supplementary information, Supplemental Figure S1. When examined across all animals, this trend emerged statistically. Specifically, mean left ventricular end-diastolic volume (EDV) decreased from baseline to class 1 hemorrhagic shock (96.4 vs. 59.3 ml, *p* < 0.0001; Figure 4B), and, overall, the class of hemorrhagic shock was associated with decreasing EDV (*p* < 0.001; Figure 4B). When accounting for multiple comparisons, however, there was no difference detected between neither class II and III (*p* = 0.46), nor class III and IV (*p* = 0.95) hemorrhagic shock. Subjectively, however, the EDV trend followed the logarithmic hemorrhage trajectory, which front-loaded the bleed (decreasing logarithmically) (Figure 4B; black line, right axis).

Systemic vascular resistance (SVR) was used as a measure of afterload and was compared between each class of hemorrhagic shock. Overall, SVR was associated with the class of hemorrhagic shock, where each class of hemorrhagic shock was associated with an increase in SVR (*p* < 0.001; Figure 4C). When accounting for multiple comparisons, the baseline period was statistically different from all classes of hemorrhagic shock (*p* < 0.001). Class I significantly differed from classes III and IV (*p* < 0.0001). Class II was also significantly different from class IV (*p* < 0.0001).

Preload recruitable stroke work (PRSW) was also associated with class of hemorrhagic shock (*p* < 0.001; Figure 4D). When accounting for multiple comparisons, PRSW was significantly increased between baseline and class 1 of hemorrhagic shock (*p* < 0.0001). Here, this load-independent measure of inotropy was significantly decreased from baseline to class III and class IV of hemorrhagic shock.

### Coronary flow during hemorrhagic shock

**FIGURE 5:**
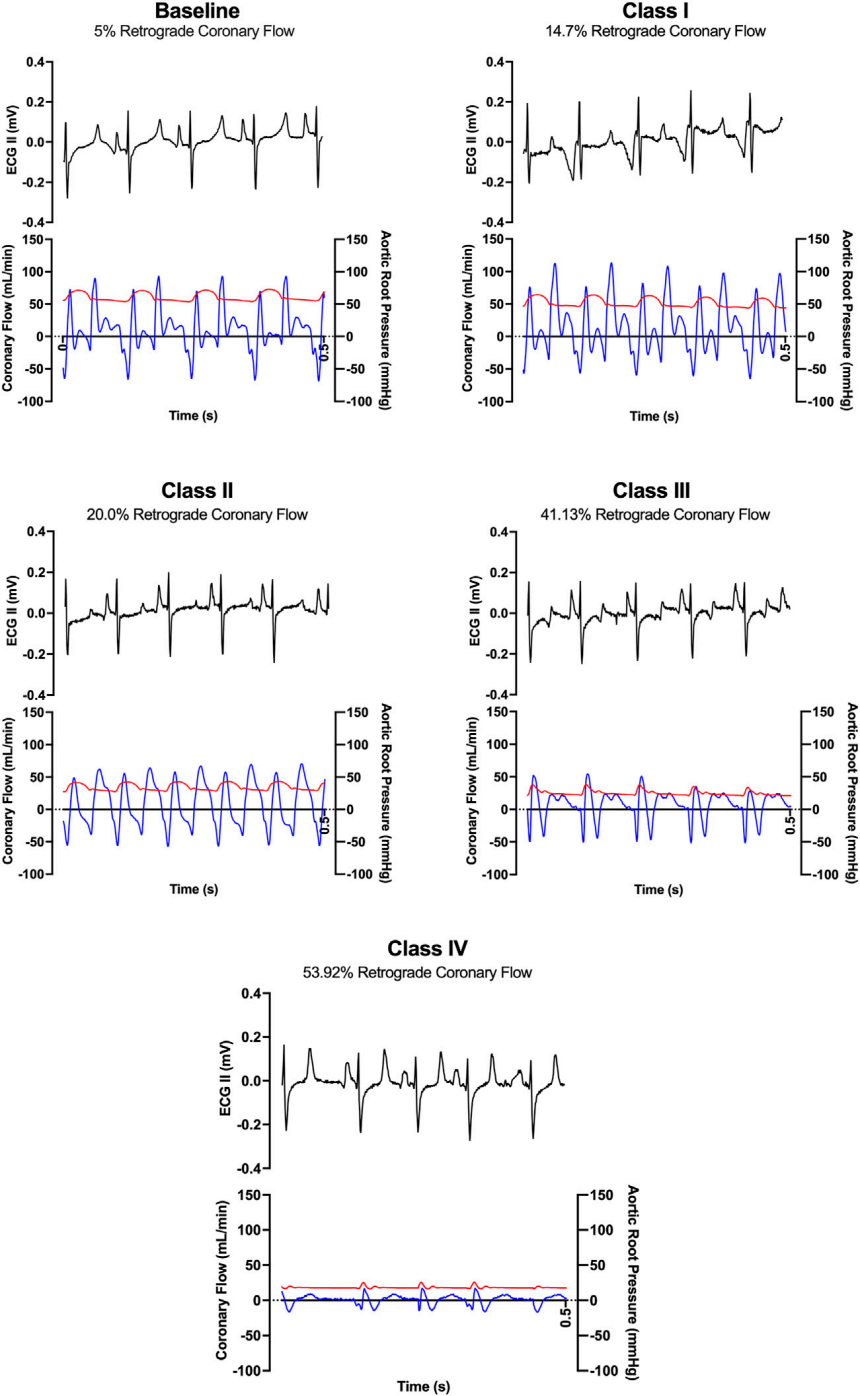
Representative ECG tracings (black) and coronary flow tracings (blue) superimposed on aortic pressures (red) over time (0.5 s) from one animal during baseline and each class of hemorrhagic shock (I-IV).

**FIGURE 6:**
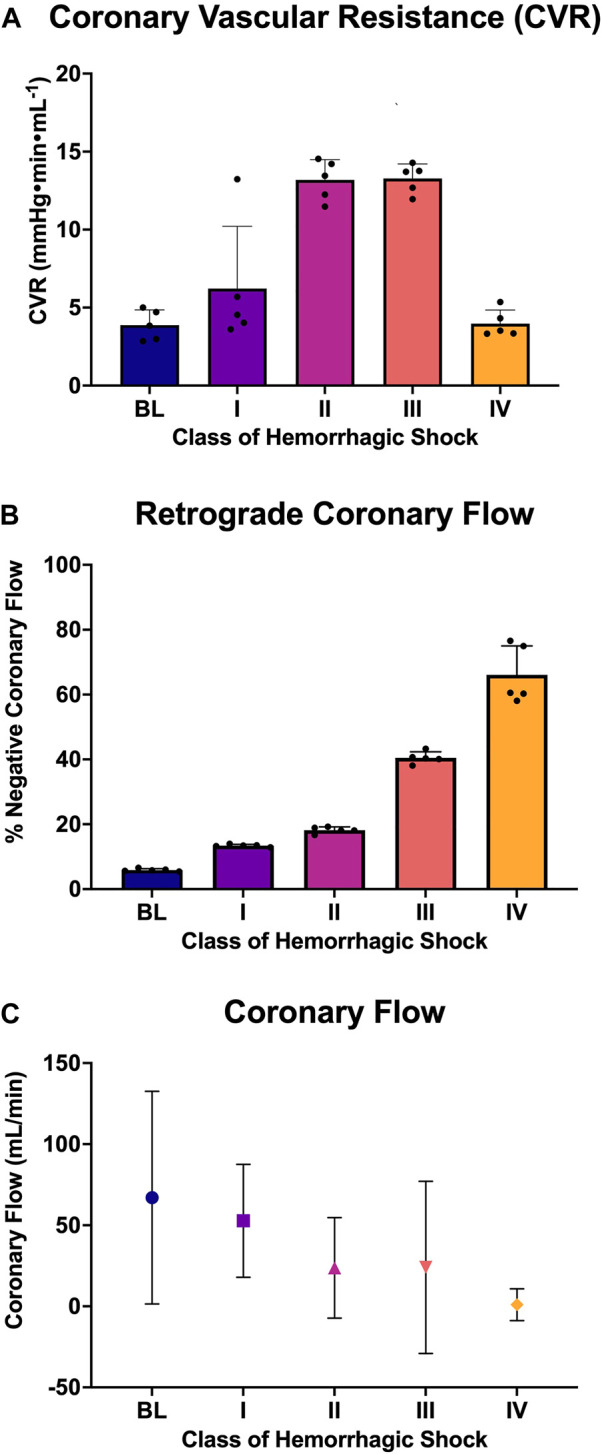
Analysis of coronary flow: **(A)** Coronary vascular resistance during diastole, **(B)** the percent of retrograde coronary flow during diastole at each class of hemorrhagic shock (I-IV), and **(C)** the coronary flow (mL/min; mean and standard deviation) at each class of hemorrhagic shock (I-IV).

LAD coronary flow data were examined for each animal, alongside telemetry and waveform data. One such animal’s representative coronary flow tracings are shown for each class of hemorrhagic shock (Figure 5; blue lines) along with aorta pressure (red line) and ECG (black line). Qualitatively, as animals progress from the baseline into worsening hemorrhagic shock, their peak coronary flows become lower, their total (area under the flow curve) decreases, and the amount of time spent with the retrograde flow (i.e., in flow reversal) increases. Coronary vascular resistance (CVR) was examined and found to increase with the class of hemorrhagic shock, except during class IV of shock (Figure 6A). It differed significantly between baseline and class II and III (*p* < 0.05).

When all animals were examined statistically, the amount of diastole spent in flow reversal (percent of the length of diastole) was associated with the class of hemorrhagic shock (Figure 6B), with baseline: 5.6%, class I: 13.55%, class II: 18.22%, class III: 41.1%, and class IV: 63.9%**.** Here, when accounting for multiple comparisons, retrograde flow differed significantly from baseline and class II, III, and IV (*p* < 0.0001; Figure 6C). It also differed significantly between classes I and II and classes III and IV (*p* < 0.0001). Class III differed significantly from class IV (*p* = 0.026). Overall, a dose-response is seen between the proportion of diastole spent with the retrograde flow and the class of hemorrhagic shock.

## Discussion

The goal of this study was to quantify coronary flow reversal alongside LV function as swine progress from baseline through the four classes of hemorrhagic shock. We showed an association between increasing severity of shock and increasing proportion of diastole spent in flow reversal, alongside the LV functional deterioration, which was quantified using LV PV loops. We also demonstrated an association between load-independent inotropy (PRSW) and states of severe hemorrhagic shock–meaning that the LV functional deterioration seen during class III or IV shock may be more severe than one would expect from the preload and afterload influences alone. Overall, as animals progressed from baseline through worsening classes of hemorrhagic shock, their proportion of time and total diastolic flow reversal increases as the LV function deteriorates and, in severe hemorrhagic shock, load-independent inotropy drops.

Few studies have described the relationship between hemorrhagic shock and coronary flow. Quantifying this relationship, however, alongside left ventricular function is becoming increasingly important as directed coronary flow augmentation may prove to be an important therapeutic target. In prior work, we demonstrated that, compared to baseline, hemorrhagic shock was associated with a reduction ESP and ESV, as well as SW, elastance, and contractility. Eight Much of this was known, at least in part, because these are preload-sensitive metrics of LV function. We further demonstrated, however, that shock was associated with increased time (% of the cardiac cycle) spent in coronary flow reversal, as well as the relationship between the reduction of peak coronary flows and total coronary flows. Stonko et al., 2022a We did not, however, examine coronary flow and LV function at each class of hemorrhagic shock and did not examine diastole in particular, which is the critical period in which the LV receives flow. These data extend this understanding to the progression between pre- and post-hemorrhage to the spectrum of hemorrhagic shock and establish a sort of dose-dependent relationship between the class of hemorrhagic shock and the percentage of time of diastole spent in retrograde coronary flow. Prior work only evaluated pre- and post-bleed, and because the coronary flow findings were unexpected, it was unable to rigorously evaluate these effects at each class of shock, nor was it able to carefully align the class of shock, coronary flow, and LV function all together in time. The diastolic analysis is especially important because as forward flow is reduced during diastole, smaller total volumes of blood reach the myocardium. This, in turn, impacts the contractility of the heart, as our data shows that inotropy decreases with the loss of blood volume. Though this is expected from the reduction of preload and afterload, there is also a reduction in PRSW, which is purportedly preload and afterload independent. Moreover, PRSW was similar but increased between baseline through class I hemorrhagic shock, reversing the trend than from class II-IV shock. This is not surprising because as hemorrhage is commenced, there is an expected early compensatory intrinsic catecholaminergic response which is expected to increase PRSW independent of preload (Feneley et al., 1992; Inuzuka et al., 2016). Because PRSW is a load-independent measure of inotropy within physiologic preload/afterload conditions, it follows that it would increase in class I shock as part of this response. Then, as the compensatory response tapered over time and as the animals fell further outside normal physiologic preload states and the intrinsic catecholamine response became less important relative to load, PRSW decreased with each subsequent class of shock. This relationship between class of shock, coronary flow and PRSW is probably also confounded by anemia secondary to blood loss and loss of oxygen carrying capacity. Future experiments may be designed to quantify the relative effect of these contributors.

Most research on coronary flow was performed in the 1940s and 1950s in canine models. In the study by Sarnoff and Berglund (1954), the coronary flow was manipulated, and its effects on left atrial pressures were quantified. It established a relationship between decreased coronary flow and myocardial failure. However, it did not examine coronary waveform flow changes across the spectrum of hemorrhagic shock nor alongside PV loops. Other research looked at changes in coronary flow but, more specifically, how these changes impact myocardial metabolism and oxygen consumption (Edwards et al., 2021b). The study did not look at the direction of coronary flow nor its variation within the cardiac cycle, but only its total magnitude. Another study by Opdyke and Wiggers (1946) used an invasive flow meter to measure coronary flow. While they also measured the total flow, its intra-cardiac cycle direction was never quantified, and the method of flow measurement was unreliable. Our study focused on gaining a quantitative analysis of coronary flow response to hemorrhagic shock. We also couple PV loop analysis to quantify left ventricular function and changes in cardiovascular physiology resulting from decreased myocardial perfusion at the same time point as the coronary flow is assessed.

Our current study has significant implications for resuscitation practice; coronary flow can be used as a therapeutic target for clinical techniques and next-generation left-sided resuscitation strategies. While this may be obvious at face value, current resuscitation techniques focus on intravenous blood delivery or right-sided resuscitation. Giving volume to the right side of the heart does not guarantee it will be pumped efficiently to the left side and into the systemic circulation. Our study data has shown that as hemorrhagic shock progresses, perfusion of the heart decreases in hand with LV function, even beyond the effect expected within the bounds of the ESPVR and EDPVR; PRSW is also affected. This creates a well-established spiral punctuated by exsanguination cardiac arrest: with less blood being pumped, less blood will reach the left side of the heart.

The observation that coronary flow deteriorates and reverses during hemorrhagic shock may provide a specific target for intervention. Specifically, would maintaining forward coronary flow during diastole alone affect outcomes in hemorrhagic shock? This is important because left-sided resuscitation through methods such as REBOA, ECMO, and selective aortic arch perfusion (SAAP) to are already known to augment coronary artery flow through each of their established mechanisms and to different extents. Our group has previously demonstrated the importance of passive afterload support in the form of REBOA and its impacts on maintaining the directionality of coronary flow. Stonko et al., 2022a; Cannon et al., 2018; Khan and McMonagle, 2019; Vella et al., 2019; Wasicek et al., 2019; Stonko et al., 2022e REBOA, however, is contingent on spontaneous circulation. To achieve spontaneous circulation during ECA, the current practice is to perform CPR with a REBOA in place, but previous studies have shown that the use of CPR is terrible for brain perfusion (Abdou et al., 2022; Patel et al., 2022b; Edwards et al., 2022). Therefore, other methods, such as SAAP and ECMO, may be better alternatives to CPR in the absence of spontaneous circulation. SAAP, in particular, theoretically overcomes both sides of the spiral: it provides passive afterload support by balloon occlusion like REBOA, but by facilitating left-sided perfusion can also directly overcome the targetable effect described here with coronary perfusion sans open or closed compressions, along with the rest of the aortic arch including cerebrovascular circulation which is just, if not more, important to long term patient outcomes. (Edwards et al., 2021b; Patel et al., 2022b; Barnard et al., 2017; Madurska et al., 2020) When the data presented here is paired with previous work Stonko et al., 2022e showing that cerebrovascular blood flow is reduced in shock and not rescued with cardiac massage, these point to a need for a therapeutic strategy that augments afterload and focuses resuscitation on the coronary arteries to achieve ROSC and also the CNS to preserve neurologic function until ROSC can be established. These two papers motivate next-generation resuscitative strategies for exsanguination cardiac arrest from the left side of the heart. SAAP may provide a therapeutic maneuver to address the established need for improved CNS flow, as well as address the coronaries.

There are many limitations to this study. A non-linear model of exsanguination was employed for all animals, which does not reflect how hemorrhage occurs across the breadth of human exsanguination pathologies. Furthermore, to interpolate hemoglobin to calculate myocardial oxygen consumption, then, we used a non-linear regression model to derive hemoglobin measures at each class of shock from pre- and post-hemorrhage hemoglobin values. This may have induced a small estimation error compared to, say, a linear model to estimate hemoglobin values at exact timepoints. The coronary flow term’s effect on the DO2 computation was, however, an order of magnitude more important than the hemoglobin term let alone a small estimate difference. Once hemorrhage commenced, there were of course rapid changes in animal physiology which require close attention. For this reason, we limited additional instrumentation, such as a right heart catheter or biventricular PV catheters, or even frequent other blood samples and monitoring which may have given us broader data to interpret. This was done to prioritize the focus on data capture and fidelity on our primary outcomes of interest. This produced a relatively narrow and focused study on coronary flow and LV function but that limited confounders that may have been introduced from other low-priority questions. Indeed, during this study we observed left coronary flow reversal during systole, but we focused on the implications during diastole because that is when the heart is being perfused. Though flow reversal during systole may also be important and a target of intervention during hemorrhagic shock, the implications are less clear than diastolic flow reversal; also, coronary flow reversal during systole is an observed phenomena in multiple pathologies, and even in small coronaries in the absence of pathology (Voci et al., 2004). Furthermore, while swine are used extensively in cardiovascular research, there are still physiologic differences. It, however, would be challenging and unethical to instrument human patients with coronary flow probes or PV loops when they are in hemorrhagic shock to capture this relationship more directly. Related to this, though the PV loops are minimally invasive catheter-based technologies, they do cross the aortic valve. Though the animals have to our knowledge, no cardiac pathology, this may affect coronary flows to some extent if there is stenting open of the aortic valve during diastole, which also affects diastolic coronary filling. Furthermore, swine, like humans, exhibit a cardiac suppressive effect in response to isoflurane, especially longer exposures to isoflurane. Fortunately, this study was relatively short but did include exposure to isoflurane, whereas a human exposed to trauma or bleeding in the field they would probably not be intubated and on isoflurane, so this is not perfectly analogous to a field model of human hemorrhage. Future work may investigate coronary flow throughout the cardiac cycle using indirect (e.g., ultrasound) or right heart-based measurements.

## Conclusion

Hemorrhagic shock is associated with diastolic coronary flow reversal with a dose-dependent relationship between the severity of shock, and the proportion of diastole where flow is reversed. Though this trend is seen as preload and afterload drop during hemorrhage, there is also a progressive decrease in load-independent inotropy (preload recruitable stroke work) alongside the other LV and coronary flow changes known to change in severe hemorrhagic shock; though correlated this may not be causative and it is likely multifactorial. Nevertheless, these data suggest an important role for left-sided, coronary artery-directed resuscitation methods following exsanguination cardiac arrest to provide afterload support and counteract the rise in retrograde coronary flow and decrease in contractility.

## Data Availability

The raw data supporting the conclusion of this article will be made available by the authors, without undue reservation.
